# Downregulation of promoter methylation gene PRDM5 contributes to the development of tumor proliferation and predicts poor prognosis in gastric cancer

**DOI:** 10.7150/jca.59998

**Published:** 2021-10-03

**Authors:** Jing-jing Teng, Wen-jing Zhao, Xun-lei Zhang, Da-Kun Zhao, Xin-Yue Qiu, Xu-dong Chen, Lei Yang

**Affiliations:** 1 Department of Oncology, Affiliated Tumor Hospital of Nantong University, No.30 Tongyang North Road, Nantong 226361, China.; 2 Cancer Research Center Nantong, Tumor Hospital Affiliated to Nantong University, Nantong.; 3 Department of Pathology, Tumor Hospital Affiliated to Nantong University, Nantong, China.

**Keywords:** GC, Promoter methylation, PRDM5, proliferation, prognosis

## Abstract

**Background:** Epigenetic aberrations of tumor suppressor genes (TSGs), particularly DNA methylation, are frequently involved in the pathogenesis of gastric cancer (GC). Previous studies have shown that PRDM5 is methylated and silenced in GC. However, the role of PRDM5 in GC progression has not been explored.

**Methods:** The expression and epigenetic alterations of PRDM5 in GC were analyzed in public datasets. The mRNA and protein expression of PRDM5 in fresh tissues were detected by semi-quantitative PCR and Western blot. And expression of PRDM5 in gastric paracarcinoma and carcinoma tissues from 162 patients was detected by immunohistochemistry (IHC) and assessed the association with different clinicopathological features. The prognostic value of PRDM5 in GC patients was evaluated using Kaplan-Meier plotter. We also studied promoter region methylation of PRDM5 in GC by methylation-specific PCR (MSP). The effects of PRDM5 on cell proliferation and migration were conducted by functional experiments *in vitro*.

**Results:** The expression of PRDM5 was downregulated in GC, and that was associated with poor survival and tumor progression. And PRDM5 expression was found to be an independent prognostic factor for GC. We also found that the methylation of PRDM5 promoter was closely related to the histopathological types and the progression of tumors through the public relations database. *In vitro*, ectopical expression of PRDM5 inhibited the growth of tumor cells, while knockdown of PRDM5 increased the proliferation and migration of tumor cells.

**Conclusion:** These results suggest that PRDM5 may be a novel TSG methylated in GC that plays important roles in GC development. And we found PRDM5 as a potential survival biomarker for GC, especially in well differentiated GC. PRDM5 expression was significantly correlated with tumor stage and histological type.

## Introduction

Gastric cancer remains an important cancer worldwide and it is the fifth most frequently diagnosed cancer and the third leading cause of cancer death. In particular, the incidence of GC is the highest in East Asia 1. In the past few decades, comprehensive utilization of various treatment methods has significantly improved the survival of patients with early GC, but the prognosis of advanced GC did not significantly improve 2. When GC is mostly diagnosed as advanced, the prognosis of advanced GC is usually poor, with a 5-year overall survival rate of less than 20% 3. Therefore, a better understanding of the pathogenesis and molecular events of GC can help us to establish new diagnostic techniques, treatment regimens and prevention strategies for this disease.

The occurrence of GC involves the accumulation of various genetic and epigenetic changes, which leading to the activation of oncogene and the inactivation of tumor suppressor 1. An increasing body of evidence indicates that in addition to changes in *DNA* sequence, epigenetic alterations contribute to GC initiation and progression 2, including DNA methylation of CpG islands, post-translational modifications of histones, microRNAs, noncoding RNAs, and nucleosome positioning 3. DNA methylation is the first epigenetic marker that has been proved to be closely related to tumorigenesis, and it provides a stable gene silencing mechanism. DNA methylation is associated with histone modification and other chromatin related proteins and plays an important role in regulating gene expression and chromatin structure 4. So far, a large number of genes with different biological functions have been found to be methylated in GC. Among them, PRDM5 was found to be frequently methylated and silenced in gastric cancer 5.

PRDM5 (also known as PFM2) is a member of the PRDM gene family and is usually located in the region 4q25-26 6. The PRDM5 protein was first identified from an EST database based on its N-terminal conserved PR domains, which typically have sequence-specific DNA binding activity 7. Epigenetic silencing of PRDM5 is a common event in GC, and it has been demonstrated that this silencing of PRDM5 is mediated by DNA methylation or H3K27 trimethylation 8. Moreover, methylation of PRDM5 can be detected in gastric juice of early GC 9, this suggests that PRDM5 is expected to be a good indicator for the diagnosis of GC. PRDM5 has been shown to inhibit tumor growth *in vitro* in patients with advanced GC, but its specific clinical significance and the critical roles in GC have rarely been probed.

In this study, we verified that the silencing of PRDM5 in GC was due to the methylation of its promoter, and confirmed its tumor suppressive effect in GC. We further explored the clinical significance of PRDM5, suggesting that the silencing of PRDM5 expression is closely related to clinical staging of GC and can indicate a poor prognosis.

## Materials and methods

### Public cancer database analysis

The expression analysis of PRDM5 in GC specimens was based on data retrieved from The Cancer Genome Atlas (TCGA) data portal (https://portal.gdc.cancer.gov/). R software (http://www.r-project.org/) was applied to extract and normalize the mRNA expression data from these databases, and then the statistical analyses were performed using GraphPad Prism software version 8.0 (GraphPad Software Inc.). To explore the molecular mechanism contributing to the aberrant expression of PRDM5 in gastric cancer, the correlations between mRNA expression of PRDM5 and DNA methylation were analyzed using online database MEXPRESS (https://mexpress.be) 10.

### Tumor samples

This study was approved by the Medical Ethics Committee of the Affiliated Tumor Hospital of Nantong University. A total of 162 formalin-fixed paraffin-embedded (FFPE) specimens and fresh surgical tissue samples were used in this study. After the review and approval of the Ethics Committee of the Affiliated Cancer Hospital of Nantong University, 162 patients with gastric cancer who were treated in the hospital from January 2007 to December 2013 were selected as the research objects. Tissue specimens of all selected cases were selected according to the following criteria: 1) The selected cases were based on relevant imaging diagnostic data; 2) All patients had clear postoperative pathological diagnosis; 3) No preoperative treatment, including chemoradiotherapy, targeted therapy and immunotherapy; 4) Classification according to WHO (2008) standards; 5) All enrolled gastric cancer patients had complete and detailed clinicopathological data, and the follow-up time was up to 2020-08-12. The tissue samples were collected and made into tissue microarray. Specifically, the specimens were GC tissue and paired adjacent normal tissues (>5 cm from the corresponding tumor edge). No patient received any adjuvant therapy before surgery. Clinicopathological information was also collected. Written informed consent was acquired from all human participants after complete description of the study. All procedures performed in studies involving human participants were in accordance with the ethical standards of the institutional and/or national research committee and with the 1964 Helsinki Declaration and its later amendments or comparable ethical standards.

### Immunohistochemistry

In the immunostaining of the tissue microarray (TMA), the primary antibody was anti-PRDM5 antibody (1:300 dilution, number: 7D4C12, NOVUS). The specific experimental procedures refer to the previous literature 11. The pathologists who were unaware of the patient's data evaluated the two points selected by the same patient's gastric cancer tissue. When the scores were inconsistent, the original wax block of the patient was found to be re-scored to determine the final scoring results. Scoring rules: The percentage of positive cells in each field was used as the scoring basis, that is, the score was 0-100 points. During the analysis, we determined that the score less than 25 was divided into the low-expression group, and the score greater than or equal to 25 was divided into the high-expression group.

### Cell culture and transfection

We selected three gastric adenocarcinoma cell lines (AGS, SGC-7901, and HGC-27), among which HGC-27 is an undifferentiated low-adhesion GC cell. They are provided from Center Laboratory of Tumor Hospital affiliated to Nantong University. All cell lines were grown in RPMI-1640 supplemented with 10% FBS (Invitrogen, Carlsbad, CA) at 37°C and 5% CO_2_. Small interfering RNAs (siRNA) targeting the PRDM5 transcript were purchased from ObiO Technology. Sequences were (5'-3'): AGCUAAAACGUCAUAUGAUTT and AUCAUAUGACGUUUUAGCUTT. The siRNA transfection was performed using Lipofectamine 2000 (Thermo Fisher Scientific, Inc., Waltham, MA, USA) according to the manufacturer's instructions. The culture medium was replaced following a 4‑h transfection. After 48 h, the cells were harvested for subsequent experiments. For PRDM5 knockdown, the cells were transfected with 50 nM specific PRDM5 siRNAs or scrambled siRNA using serum-free Opti MEM (from Thermo Scientific). Small activating RNAs (saRNA) targeting the PRDM5 transcript were purchased from Shanghai Genechem. Sequences were (5'-3'): GCCCGGATCCGTTCCTGCCAT and ATGGCAGGAACGGATCCGGGC. The specific experimental process is completed by referring to the product manual.

### Cell proliferation assay and colony formation assays

Treated cells (1 × 10^6^ for AGS and SGC7901) were seeded in triplicate into 96-well plates and allowed to incubate for 24 h, 48 h and 72 h. Cell viability was assessed according to the Cell Counting Kit-8 (CCK-8) protocol (Dojindo, Kumamoto, Japan). For colony formation, treated GC cells were seeded in 6-well plates at 200 cells per well 23. Two weeks later, colonies were fixed with 4% paraformaldehyde and stained with 0.5% (W/W) crystal violet (diluted in phosphate buffer saline, PBS) for 30 min.

### Cell migration assays

Transwell cell migration assays were performed using boyden chambers with a polycarbonate Nucleopore membrane. 5 × 10^4^ cells in 200 µl serum-free Dulbecco's modified Eagle's medium were placed in the upper part of each chamber and the lower compartments were filled with 500 µl Dulbecco's modified Eagle's medium containing 10% serum as described previously.

### Western blot analysis

Total protein was isolated from the tissues by using RIPA buffer; 100 μg protein were loaded and separated by 10 %SDS-PAGE and transferred to PVDF membranes (Millipore, Billerica, MA, USA). Next, the membranes were incubated with specific antibody for PRDM5 (Abcam, 1:1500) or GAPDH (Abcam, 1:5000) at 4 °C o/n. The membranes were washed and then incubated with secondary antibody for 2 h at room temperature. Finally, the membranes were developed using ECL kit (Pierce, Rockford, IL, USA) and exposed to X-ray film for analysis by Image.lab3.0 software.

### qRT-PCR

Total RNA was extracted from the tissues using TRIzol (Invertrogen, USA) following the manufacturer's manual. cDNA was synthesized by reverse transcription using RT kit (Promega, Madsion, WI, USA) following the manufacturer's manual. PCR was performed with Taq Master Mix (Promega, Madison, WI, USA) with the primers: PRDM5 GATCAAGTGGGTGCTCACAA and CATTGATAGGGACGCTCACC, product 474 bp, CAC GATGGAGGGGCCGGACTCATC and TAA AGACCTCTATGCCAACACAGT, product 225 bp. Amplification conditions were as follows: 5 min at 94 °C (one cycle); 30 s at 94 °C, 30 s at 58 °C, and 30 s at 72 °C (35 cycles) and 72 °C for 5 min (one cycle).

### Methylation-specific PCR (MSP)

Genomic DNA was extracted from the tissues using Universal Genomic DNA Extraction Kit (Takara, Tokyo, Japan). Genomic DNA (1 μg) was modified with sodium bisulfite using EZ-DNA methylation kit (Zymo research, Orange, CA, USA). Bisulfite-treated DNA was used for methylation-specific PCR (MSP). MSP primers were designed by online software (http://www.urogene.org/methprimer/index1.html). The primers sequence were as follows:

- Methylation primer: TTTTATAGGGAGTAATGGTTTAGCG and GCTAATTAACCCGAAATTAACGAC;
- Unmethylation primer: TTTATAGGGAGTAATGGTTTAGTGG and CACTAAT TAA CCCAAAATTAACAAC.

PCR amplification system (25 µl) includes 10× Buffer 2.5 µl, dNTP 1.0 µl, 1 µl each methylation or unmethylation primers, DNA template 2 µl, and MgCl_2_ 2 µl. PCR parameters include 95 °C for 5 min, then 95 °C degeneration for 30 s, annealing for 30 s, and 72 °C extensions for 30 s. PCR products were electrophoresed on 2% agarose gel, and the images were scanned using the UV gel imaging system.

### Statistical analysis

The statistical analyses were performed using GraphPad Prism software version 8.0 (GraphPad Software Inc.) and SPSS version 25.0 (SPSS Inc.). Differences between groups were determined using the Chi-square test or Student's t test. Overall survival (OS) after surgery was calculated using the Kaplan-Meier method. Cox stepwise multivariate regression analysis of prognostic factors was performed. A p value of less than 0.05 was considered to be statistically significant.

## Results

### PRDM5 expression is frequently decreased in GC

**Figure 1:**
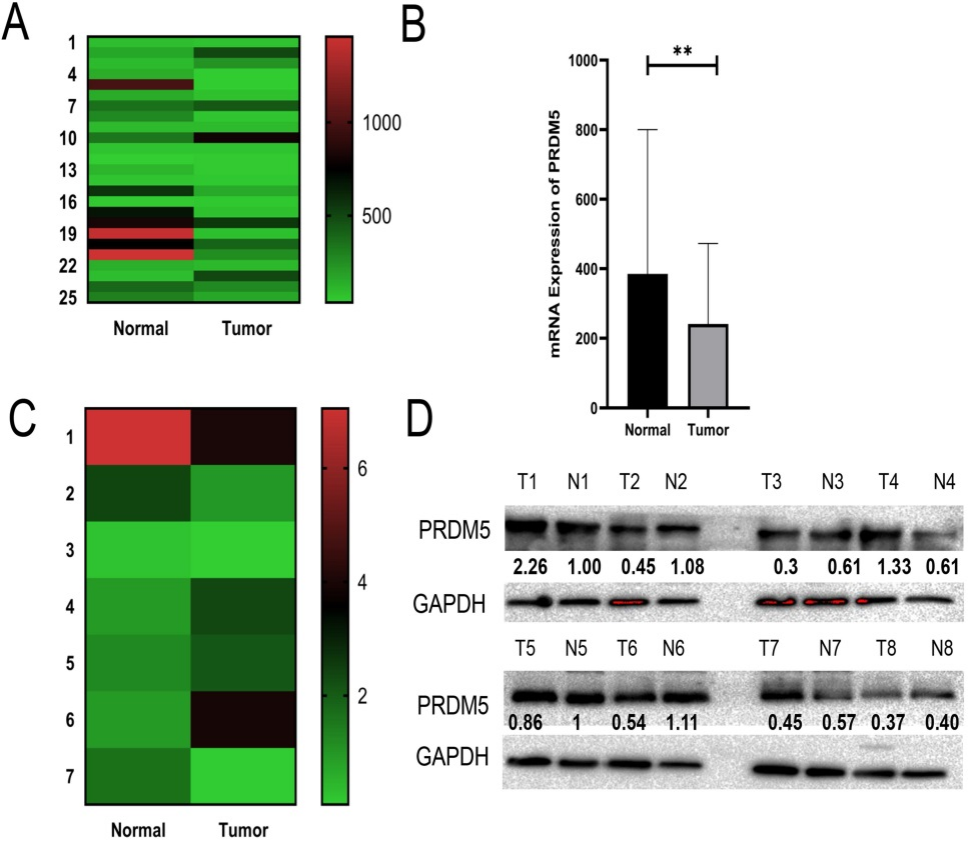
PRDM5 mRNA expression and protein expression are downregulated in GC. **A-B** Expression of PRDM5 was frequently downregulated in gastric tumor tissues (tumor) compared with adjacent or normal gastric tissue samples (normal) in public databases. **C-D** mRNA levels of PRDM5 detected by Q-rtPCR and protein levels of PRDM5 detected by western blot in tumors and adjacent tumors. Data are shown as mean ± SD *p < 0.05;**p < 0.01; ***p < 0.001, ****p < 0.0001.

Through analysis of PRDM5 expression in public datasets from GC patients, we found that PRDM5 was remarkably downregulated in GC tissues compared to normal tissues (Fig. 1A, B). To further verify this phenomenon, we randomly selected 8 fresh tumor tissues and paired adjacent normal tissues of GC patients for the detection of mRNA levels and protein levels. We found that The expression of PRDM5 in gastric cancer tissues was lower than that in paracancer tissues, and only a few GC tissues showed no significant change in the expression of PRDM5 (Fig. 1C, D).

### Low expression of PRDM5 was associated with clinical stage and was an independent prognostic factor for gastric cancer

**Figure 2:**
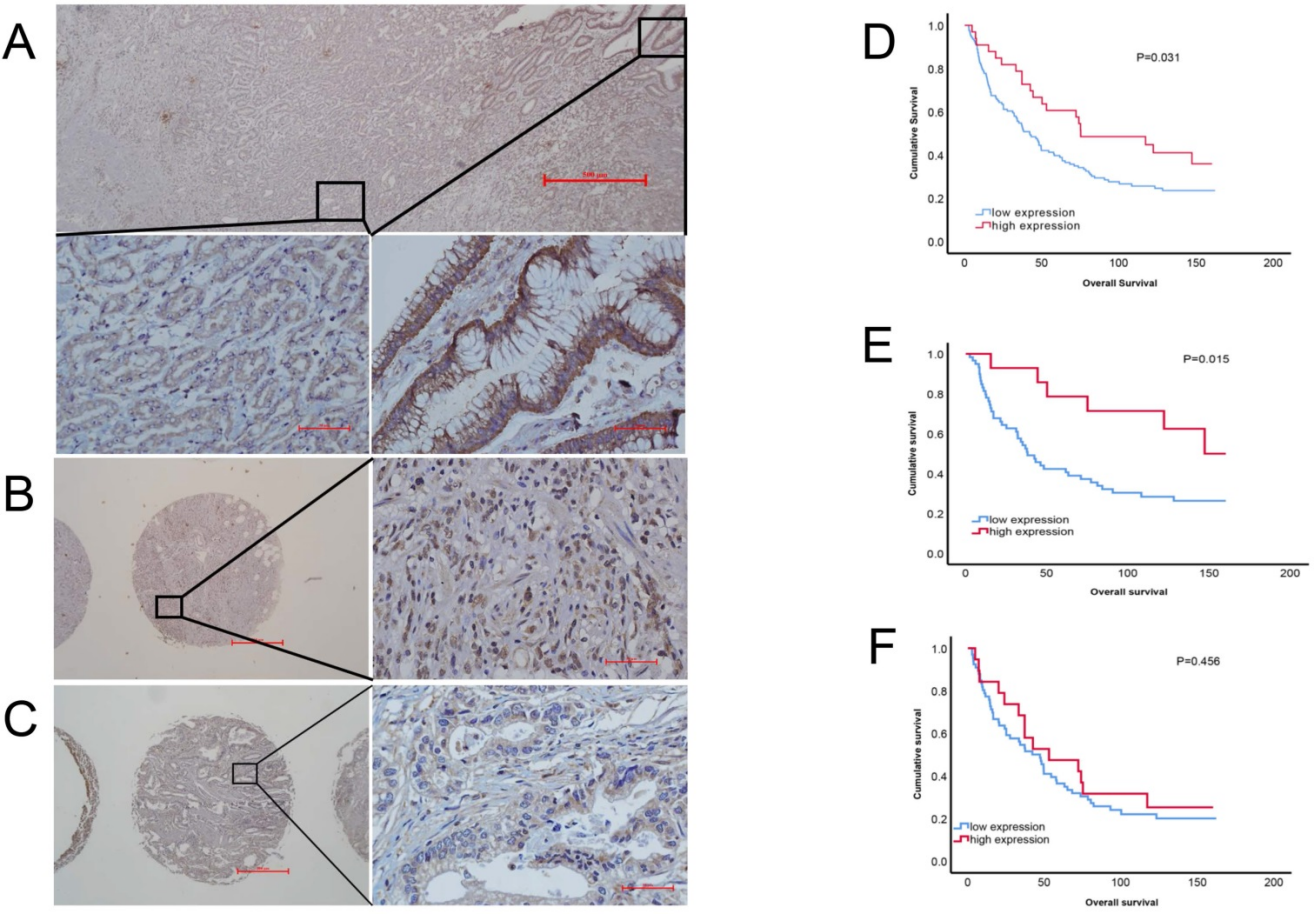
** Protein expression of PRDM5 in GC tissues (×40/×400 optical magnification). A.** PRDM5 expression in carcinoma and paracancerous tissues of a patient with GC. **B-C.** The typical examples of high and low expression in GC tissues, respectively. Original magnification ×400.** D-F.** Kaplan-Meier curves showed that PRDM5 expression is significantly associated with prolonged survival in all GC patients (**D**) and in patients with well differentiated (**E**) or poor differentiated (**F**).

To evaluate the correlation between PRDM5 expression and patients' survival, we observed RPDM5 expression that performed IHC and analyzed in 162 gastric cancer patients. According to the results of immunohistochemistry, we found that PRDM5 was generally expressed in cytoplasm, but the expression in nucleus was different. In the nucleus of gastric cancer cells, PRDM5 is rarely expressed, while in the nucleus of normal tissues adjacent to cancer, PRDM5 is almost always expressed. Representative results are shown in Fig. 2A-C. The correlations between PRDM5 protein expression and the clinicopathologic parameters of GC patients are summarized in Table 1. In GC, PRDM5 protein expression was significantly correlated with clinical stage (*p*=0.036). Kaplan-Meier curves for overall survival (OS) were analyzed. Patients with high PRDM5 expression had significantly better survival (*p*=0.031) (Figure 2D). Interestingly, we found that low PRDM5 expression in well-differentiated GC tissues suggested poor prognosis (Figure 2E), while no significant difference in prognosis was observed in poorly differentiated patients (Figure 2F). Furthermore, Univariate and multivariate Cox regression analyses were performed to identify important prognostic factors of OS (Table 2). PRDM5 expression (*p* = 0.034), tumor diameter (*p* < 0.001), clinical stage (*p* < 0.001) and vascular invasion (P=0.048) were identified as important risk factors for OS. In multivariate Cox analysis, PRDM5 expression (*p* = 0.043), tumor diameters (*p*=0.024) and clinical stage (*p* < 0.001) were found to be independent prognostic factors for OS. These findings indicate that high expression of PRDM5 could predict good prognosis in patients with GC, especially in patients with well differentiated GC.

### PRDM5 inhibited the proliferation and migration of GC cells

**Figure 3:**
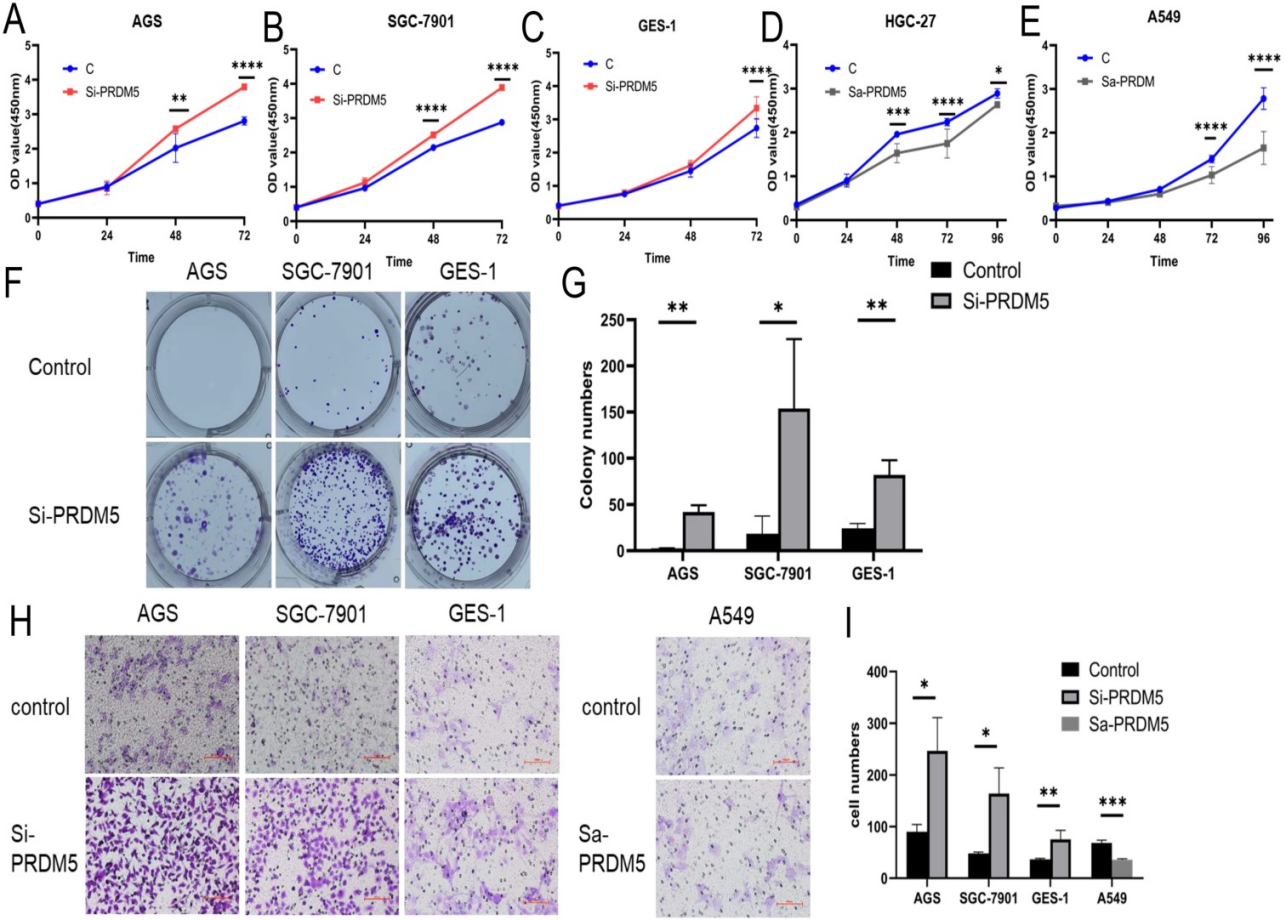
** PRDM5 inhibited the proliferation and migration of GC cells. A-E.** The proliferation ability of GC cells was inhibited by PRDM5. **F.** The proliferation ability of cells was enhanced after PRDM5 knockdown. **G.** Statistical results of cell cloning experiments. **H.** Cell migration assay after changing PRDM5 expression. **I.** Statistical results of cell migration assay.

To explore the role of PRDM5 in the development of GC, we conducted further experiments in GC cells. Firstly, we examined the basic expression of PRDM5 in three cell lines by Western blot. We found that the expression of PRDM5 in HGC-27 was significantly silenced. Next, we knocked down PRDM5 in AGS and SGC-7901 cell lines, and overexpressed PRDM5 in HGC-27 cell lines. For better quality control, gastric epithelial cell GES-1 and lung adenocarcinoma cell A549 were selected for the experiment. In A549 cell line, PRDM5 has been shown to be silenced by previous studies.Not surprisingly, we found that PRDM5 knockdown GC cells (AGS, SGC7901) significantly enhanced their proliferation ability (Figure 3A-B) and the ectopic expression of PRDM5 inhibited the proliferation of tumor cells (Figure 3D-E). After knockdown of PRDM5, the cloning and migration ability of GC cells were significantly improved (Figure 3F-H). However, after the exogenous overexpression of PRDM5 in A549, the cell migration ability was inhibited. In gastric epithelial cells GES-1, PRDM5 knockdown also significantly improved the cell proliferation, cloning and migration ability (Figure 3C, F & H).

### The low expression of PRDM5 is related to promoter methylation

**Figure 4:**
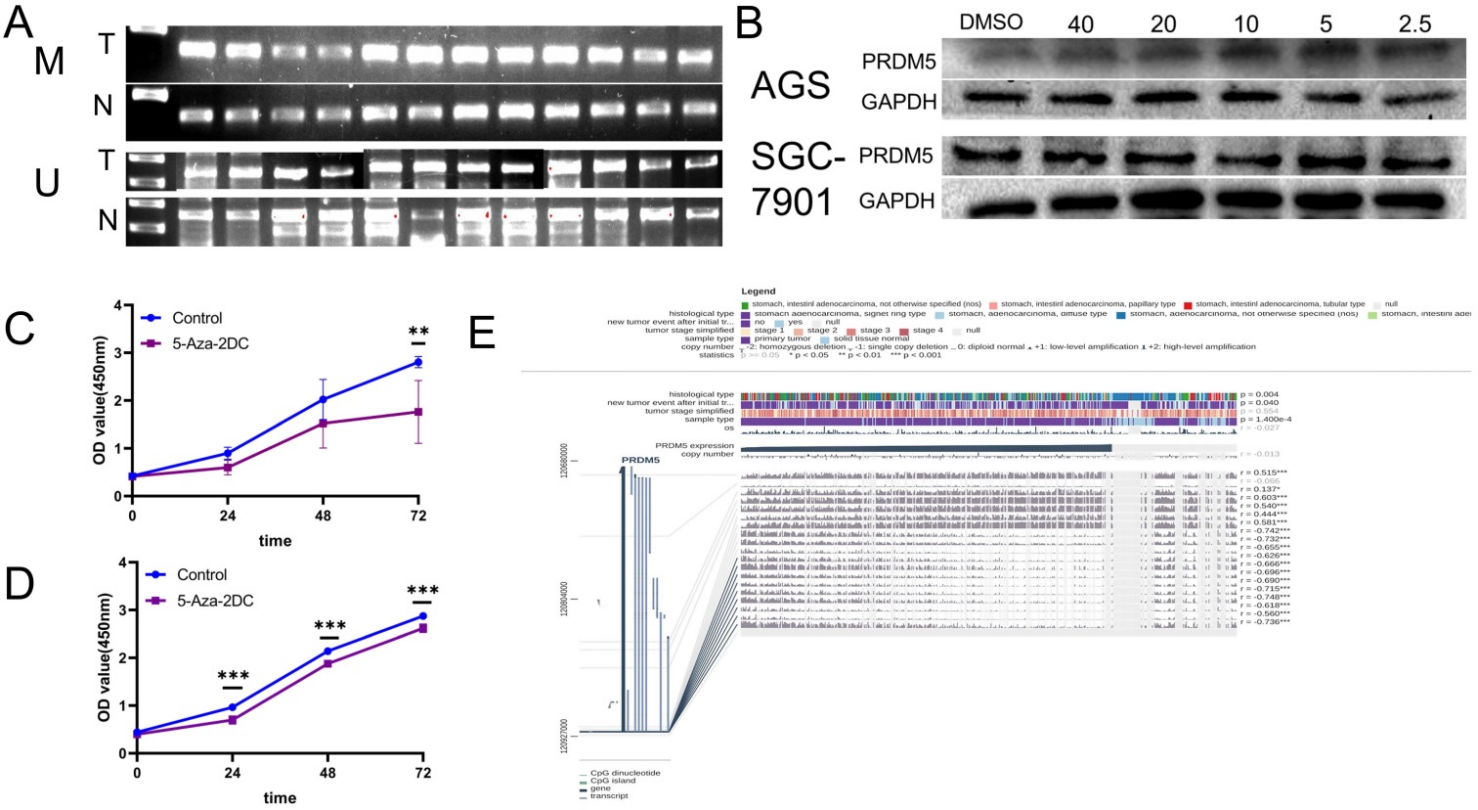
** The promoter of PRDM5 is often methylated. A.** The methylated specific primers and unmethylated specific primers were used to detect the difference of methylation between tumor and normal tissues. M primers specific to methylated template DNA, U primers specific to unmethylated template DNA, T tumor, N normal. **B.** PRDM5 protein expression in cells treated with methylation inhibitors at different concentrations (µM). **C-D.** Proliferation ability of AGS(**C**) and SGC-7901(**D**) cells which were treated with methylation inhibitors at appropriate concentrations. **E.** MEXPRESS analysis about the correlation between DNA methylation of CpG islands in the 5′ promoter region and expression level of PRDM5.

Previous studies have shown that PRDM5 is silenced in tumors due to methylation of its promoter 5. Firstly, MSP was detected in fresh GC tissues and paired adjacent normal tissues from 12 patients. We found that methylated bands could be detected in all carcinomas, but rarely in the paired adjacent normal tissues (Figure 4A). Further, we verified the methylation mechanism of PRDM5 in GC cells. By treating GC cells with different concentrations of methylation inhibitors (5-Aza-2DC), we found that suitable concentrations of 5-Aza-2DC could restore the expression of PRDM5 (Figure 4B). Next, we continued to verify the proliferation ability of GC cells after the addition of inhibitors. We found that the proliferation activity of GC cell lines was inhibited when treated with a certain concentration of inhibitor that could restore PRDM5 protein expression (Figure 4C, D). Finally, we used MEXPRESS to analyze the correlation between DNA methylation of CpG islands in the 5′ promoter region and expression level of PRDM5. The samples shown in Figure 4E are ordered by expression value. The expression of PRDM5 in GC negatively correlated with the level of DNA methylation in the promoter, which was confirmed by Pearson correlation coefficients (r up to -0.748, *p* < 0.001). MEXPRESS analysis also revealed that PRDM5 mRNA expression level was much lower in GC samples than those in control samples (*p* = 1.400e-4). Moreover, we found that the methylation of PRDM5 promoter was closely related to the histopathological types of patients and the recurrence and progression of tumors. Thus, these data demonstrate that promoter DNA hypermethylation of PRDM5 may result in downregulation of its mRNA expression, which, to some extent, supports our preliminary study results.

## Discussion

Gastric cancer is the fifth most common cancer and the third leading cause of cancer death in the world, with the highest incidence in East Asia 12. High-income regions in Asia Pacific and East Asia have the highest age-standardized incidence rates, with nearly half of global cases occurring in China 13 .This urges us to understand GC through more research, so as to alleviate or even cure GC and reduce the disease burden. Early GC can usually be cured by endoscopic resection or radical surgery, but the prognosis of advanced GC is still poor, despite chemotherapy, radiotherapy, targeted therapy, immunotherapy and other therapeutic methods. So, there are many trials going on that combine immunotherapy, chemotherapy and surgery for local GC 14. On this basis, we are still actively looking for new breakthroughs in order to help patients with GC.

Genetic and epigenetic changes can participate in the occurrence and development of GC through activation of growth-promoting pathways and inactivation of tumor suppressor pathways. Moreover, in recent comprehensive studies of genetic and epigenetic changes in GC, epigenetic changes such as activation of Wnt pathways, inactivation of cell cycle regulation, and damage to mismatch repair have been more frequent than genetic changes 15. In epigenetic changes, abnormal DNA methylation of a promoter CpG Island (CGI) can continuously inhibit the transcription of its downstream genes, and tumor suppressor genes can be inactivated by this mechanism 16. The DNA demethylation drugs 5-azacytidine (azacitidine) and 5-aza-2 '-deoxycytidine (decitabine) that are clinically used in patients with myelodysplastic syndrome can restore abnormal DNA methylation 17. Multiple clinical trials involving the use of demethylating agents for solid tumors are currently under way. Several trials have shown that demethylating agents can increase chemotherapeutic sensitivity in some drug-resistant ovarian and lung cancers 18, 19. In GC, studies have verified that methylation agents have anti-tumor effects on SN38 and CDDP resistant GC cell lines 20. However, the exact epigenetic mechanism is still unclear. Furthermore, we tried to explore tumor suppressor factors that activate this mechanism.

PRDM5 is considered to have tumor suppressive activity because its promoter contains a CpG island, which is highly methylated in tumors, leading to silencing of its expression in a variety of tumors 7. Our study shows that PRDM5 is generally downregulated in GC, which is consistent with previous studies 8. Based on the analysis of GC data from the TCGA database, we found that the mRNA level of PRDM5 was down-regulated. And we found that the protein expression of PRDM5 was generally inhibited in tumor tissues. Due to the large heterogeneity of tumors, we further detected PRDM5 protein expression in 162 cases of GC in tissue microarray. It was found that the expression of PRDM5 was negatively correlated with clinical stage. Based on these results, we wondered whether PRDM5 could predict the prognosis of patients with GC, which has not been studied before. Our results showed that patients with high PRDM5 expression had a significantly better prognosis, especially in patients with well differentiated GC. Through univariate and multivariate regression analysis, we found that PRDM5 expression is the same as clinical stage and tumor size, which are independent prognostic factors of GC. These results suggest that PRDM5 plays an important role in the treatment of advanced GC, which means that we may be able to find new therapeutic targets in the treatment of advanced GC. Therefore, we further explored the mechanism of PRDM5 in GC. As we have seen, the proliferation activity and migration ability of GC cells were increased after knockdown of PRDM5 expression, while the proliferation activity was inhibited after overexpression of PRDM5. Then, we found that PRDM5 expression was increased more or less in cells treated with methylation inhibitors. We were curious to see if methylation of the PRDM5 promoter had any clinical significance. We performed an analysis of the gene promoter methylation in a public database. We found the expression of PRDM5 in GC negatively correlated with the level of DNA methylation in the promoter. Moreover, we found that the methylation of PRDM5 promoter was closely related to the histopathological types of patients and the recurrence and progression of tumors. Thus, these data demonstrate that promoter DNA hypermethylation of PRDM5 may result in downregulation of its mRNA expression, which, to some extent, supports our preliminary study results.

Previous studies have demonstrated that promoter reporting activity of cyclin D1 (CCND1), a target gene downstream of the Wnt/β-catenin signaling pathway, is significantly reduced when PRDM5 is overexpressed.Chromatin immunoprecipitation (ChIP) assay showed that PRDM5 can directly bind to the promoters of several oncogenes such as CDK4 and Twist1.Overexpression of PRDM5 significantly reduced the levels of active transcriptional markers H3K4me3 and acetylated histone H4 of CDK4, Twist1 promoter.In addition, many other genes are regulated by PRDM5, including oncogenes such as p53, MYC and MDM2 5. In gastric cancer, whether PRDM5 plays a role in tumor inhibition through these possible pathways still needs further verification. It is worth noting that although PRDM5 expression is reduced in most tumors, such as lung cancer, cervical cancer, prostate cancer, glioma and other tumors 21-24. However, there are exceptions. In melanoma and acute myelogenous leukemia, PRDM5 is overexpressed in tumor cells and is believed to promote tumor progression through activation of the JNK pathway 25, 26. This opposite regulatory function may be due to its special role in transcriptional regulation. Studies have claimed that it can promote and inhibit transcriptional genes according to promoters and cell types 27.

Our study suggests that methylation of PRDM5 promoter plays an indispensable role in GC. However, we still need more representative clinical samples to support our results because of tumor heterogeneity. It is well known that abnormal methylation plays a significant role in the development of GC because Helicobacter pylori infection leads to abnormal methylation 28. Therefore, we should include more clinical relevant factors, such as the history of Helicobacter pylori infection, in the follow-up of patients, which would make our results more convincing. In terms of mechanism, it is far from enough to understand the role of PRDM5 as a tumor suppressor in GC. Activated growth-promoting pathways in gastric cancer include Wnt, Akt/mTOR and mitogen-activated protein kinase (MAPK) pathways. These pathways can be activated not only by activating oncogene mutations, but also by inactivating their negative regulators. PRDM5 antagonizes the Wnt/β-catenin signaling pathway in both normal and tumor cells 5. However, GC is a complex and systemic disease, and how PRDM5 plays a role *in vivo* is still unknown. However, GC is a complex and systemic disease, and there are still many questions about how PRDM5 plays its role in the body, such as why the promoter of PRDM5 is methylated and how it is methylated.More *in vivo* studies are needed to answer these questions.

In conclusion, our results found that PRDM5 plays an important role in the development of GC. Especially in advanced GC, it has good prognostic significance.

## Conclusion

In this study, we demonstrated that PRDM5 plays a role as a tumor suppressor in GC, and its down-regulation in GC patients is partly caused by DNA hypermethylation. More importantly, we found that PRDM5 is a potential prognostic factor, especially in well-differentiated GC, where high expression of PRDM5 suggests a better outcome.

## Figures and Tables

**Table 1:** Correlation of PRDM5 in GC and clinical parameters in 162 cases

	All patients (n=162)	Low expression of PRDM5	P value
**Age**			0.685
≤60	76 (46.9%)	59 (77.6%)	
>60	86 (53.1%)	69 (80.2%)	
**Gender**			0.902
Female	51 (31.5% )	40 (78.4%)	
Male	111 (68.5%)	88 (79.3%)	
**Lauren classification**			0.524
Intestinal type	34 (21.0%)	26 (76.5%)	
Mixed type	46 (28.4%)	39 (84.8%)	
Diffuse type	82 (50.6%)	63 (76.8%)	
**Differentiation**			0.713
Well differentiated	76 (46.9%)	61 (80.3%)	
Poorly differentiated	86 (53.1%)	67 (77.9%)	
**Tumor diameter**			0.919
≤5 cm	118 (72.8%)	93 (78.8%)	
>5 cm	44 (27.2%)	35 (79.5%)	
**pT classification**			0.229
T1~3	110 (67.9%)	84 (76.4%)	
T4	52 (32.1%)	44 (84.6%)	
**pN classification**			0.317
N0	55 (34.0%)	41 (74.5%)	
N1~3	107 (66.0%)	87 (81.3%)	
**pM classification**			0.580
Absent	158 (97.5%)	124 (78.5%)	
Present	4 (2.5%)	4 (100.0%)	
**Vascular invasion**			0.590
Absent	113 (69.8%)	88 (77.9%)	
Present	49 (30.2%)	40 (81.6%)	
Nerve invasion			0.503
Absent	117 (72.2%)	94 (80.3%)	
Present	45 (27.8%)	34 (75.6%)	
**Clinical staging**			0.036*
I+II	79 (48.8%)	57 (72.2%)	
III+IV	83 (51.2%)	71 (85.5%)	

*P < 0.05.

**Table 2:** Univariate and multivariate Cox proportional hazard analyses for cancer-specific survival

Overall survival	Univariable		Multivariable	
	Hazard ratio	P	Hazard ratio	P
Differentiation (well versus poor)	0.755(0.534-1.068)	0.112	0.931(0.633-1.370)	0.717
Vascular invasion	1.449(1.004-2.092)	0.048	1.156(0.758-1.7622)	0.501
Nerve invasion	1.371(0.937-2.006)	0.104	1.193(0.772-1.845)	0.427
Tumor diameter (>5 cm versus ≤5 cm)	1.966(1.365-2.833)	<0.001*	1.601(1.065-2.406)	0.024*
Clinical staging (III+IV versus I+II)	2.725(1.909-3.888)	<0.001*	2.377(1.587-3.561)	<0.001*
PRDM5 expression (high versus low)	0.592(0.365-0.960)	0.034*	0.602(0.369-0.984)	0.043*

*P < 0.05.
